# Supplementary material for: Evaluation of Commercially Available Viral Transport Medium (VTM) for SARS-CoV-2 Inactivation and Use in Point-of-Care (POC) Testing
Source: Viruses. 2020 Oct 23;12(11):1208. doi: 10.3390/v12111208 (PMC7690900; doi:10.3390/v12111208)
Supplement: Supplementary file 1 [file viruses-12-01208-s001.zip › viruses-946932-Supplementary/viruses-946932_301020_FigureS1.pdf]

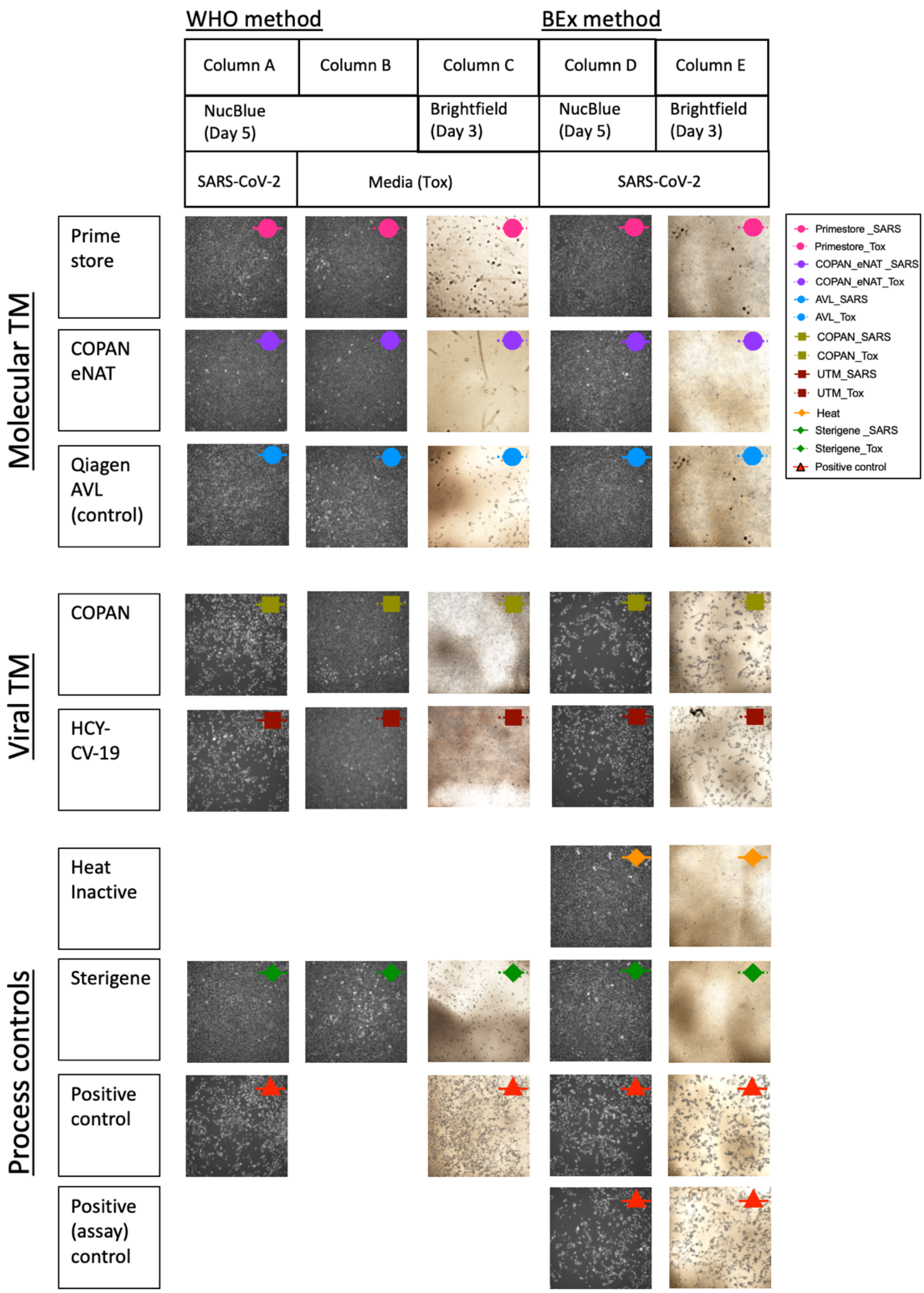

Figure S1: Images collected using the InCell 2500 HTS to count nuclei using fluorescent nuclei stain (NucBlue) and cell morphology in unstained (Brightfield) images (contrast increased in Brightfield by 80% to approximate pre-process inCell image acquisition), at 1:1000, and 1:625 dilution for the WHO, and BEx methods, respectively). Cells are represented to demonstrate toxic effect of media (d3) and final nuclei count (d5), represented in SARS-CoV-2 spiked culture (solid line) or media only (hashed line).
