# Supplementary material for: Evaluation of Commercially Available Viral Transport Medium (VTM) for SARS-CoV-2 Inactivation and Use in Point-of-Care (POC) Testing
Source: Viruses. 2020 Oct 23;12(11):1208. doi: 10.3390/v12111208 (PMC7690900; doi:10.3390/v12111208)
Supplement: Supplementary file 1 [file viruses-12-01208-s001.zip › viruses-946932-Supplementary/viruses-946932_301020_FigureS2.pdf]

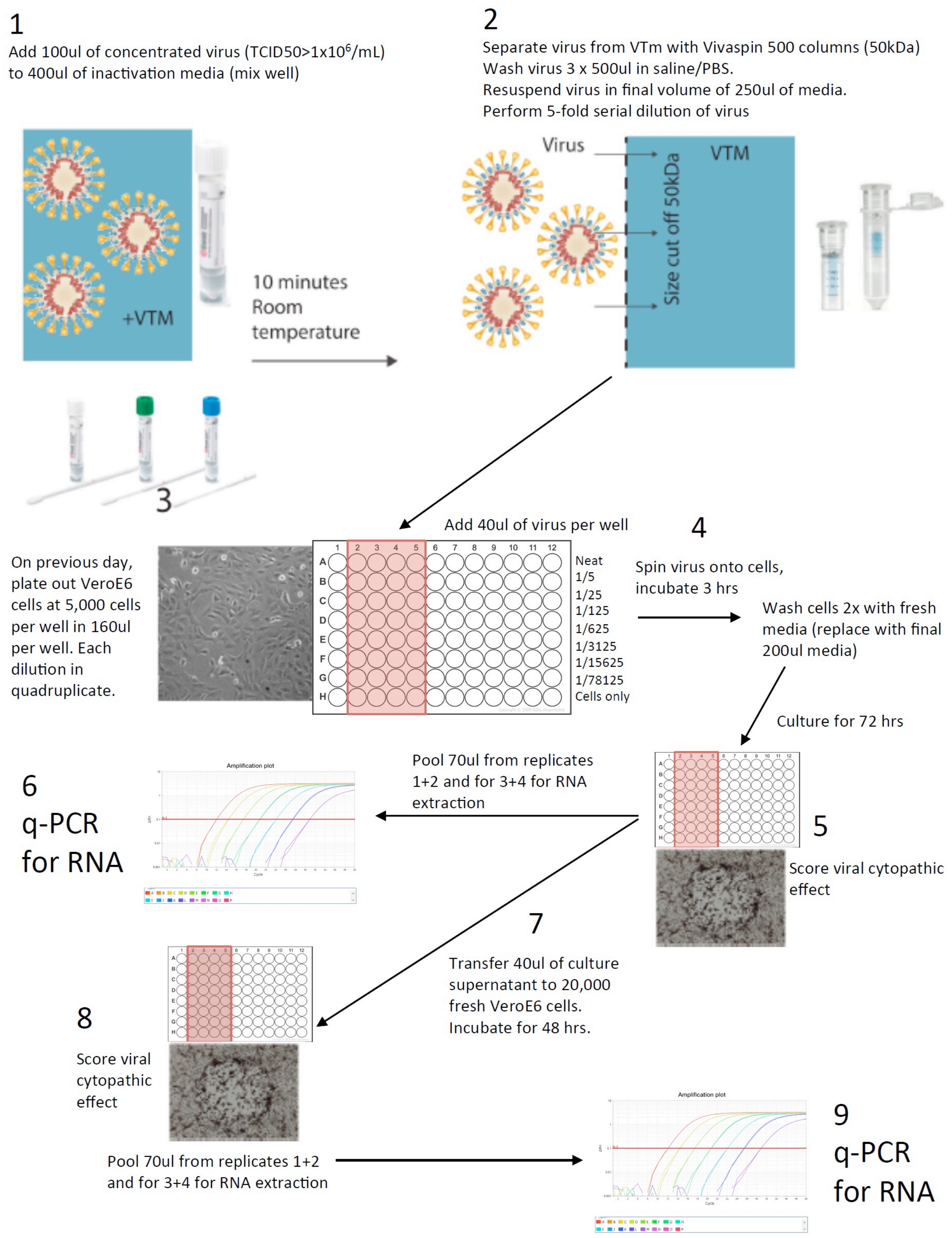

Figure S2: Schematic of SARS-CoV-2 inactivation buffer exchange (BEx) protocol using viral transport medium (VTM).
